# Translational bioethics in nursing: a conceptual review of definitions, applications and ethical implications

**DOI:** 10.1186/s12910-025-01264-8

**Published:** 2025-07-28

**Authors:** Frederick Acheampong Nimo, Abigail Gyamfi-Samakome, Esther Naana Gyan, Richard Argoh, Godson Obeng Ofori, Patience Fakornam Doe, Christian Makafui Boso

**Affiliations:** 1 Department of Adult Health, School of Nursing and Midwifery, University of Cape Coast, Cape Coast, Ghana; 2 Department of Public Health, School of Nursing and Midwifery, University of Cape Coast, Cape Coast, Ghana

**Keywords:** Concept analysis, Ethical decision-making, Interdisciplinary collaboration, Nursing ethics, Theory-practice gap, Translational bioethics

## Abstract

**Background:**

Bioethics is fundamental in healthcare, guiding ethical decision-making and patient care. Translational bioethics (TB) aims to bridge the gap between ethical theories and real-world practice, including nursing. However, the concept of TB has not been clearly examined yet. Therefore, this concept analysis was done to provide clarity and practical meaning to the concept of translational bioethics within the context of nursing.

**Methods:**

Walker and Avant’s concept analysis framework was employed to examine TB and apply it to nursing practice. A systematic search of electronic databases, including PubMed, Google Scholar, and PubMed Central, was conducted. Papers were screened and twenty-five eligible full-text records included in analysis.

**Results:**

The analysis identified four key defining attributes of TB, including bridging the theory-practice gap, ethical evaluation and decision making, interdisciplinary collaboration, social responsibility and societal impact. Antecedents included ethical dilemmas, identifying theory-practice gaps, and commitment to real-world impact. The consequences of TB encompassed improved patient care, reduction of ethical conflicts, positive social impact, and informed policy and decision making.

**Conclusions:**

This study provided a clear conceptual model of TB, offering insights into its antecedents, attributes, and consequences. Translational bioethics can be defined as an approach that bridges ethical theory and practice through research, interdisciplinary collaboration, and a focus on contextual ethical evaluation and decision making, aiming for socially responsible and impactful outcomes.

## Introduction

Translational bioethics (TB) is an emerging field of ethics that bridges the gap between biomedical research and clinical practice, highlighting how applying scientific findings to healthcare applications has ethical implications [1, 2]. Healthcare research and interventions are currently undergoing growing and dynamic ethical challenges that need to be given attention [3]. In view of that, contemporary practice requires the utilization of ethical principles in practice to meet demands of complex clinical environments, constrained resources, and evolving patient needs. For example, during the COVID-19 pandemic, a number of ethical concerns were brought to light. These included prioritization of care and equitable access, ensuring equitable distribution of resources to facilities, concerns with testing and issues pertaining to vaccine research [4]. Among healthcare professionals, nurses are always at the forefront of dealing with these ethical issues.

In fact, nurses are required to practice with high ethical standards amidst complex organizational structures and face conflicts between professional values, standardized protocols, and the demands of a busy work environment [5]. These factors contribute to the multifaceted nature of nursing practice and the array of ethical dilemmas. In view of that, nursing schools have imbibed ethics into nursing curricula to ensure students become conversant with current ethical issues in healthcare. Furthermore, many nursing councils have provided code of ethics that provide guidance on ethical nursing care. In line with this, it is imperative for nurses to fully understand new terms that emerge in the field of medical ethics so as to know how to be effectively involved and apply it to practice. The term TB has been used severally in contemporary practice but its definition and attributes have not been clearly outlined in nursing literature. It is necessary to clarify the meaning of TB to advance the science of nursing. This paper sought to perform an in-depth analysis of TB by synthetizing literature pertaining to the concept to identify its defining attributes, antecedents, consequences and empirical referents.

## Methods

### Design

The structured framework of concept analysis designed by Walker and Avant [6] was used as a guide in this analysis to understand the concept TB. The authors [6] proposed 8 steps to concept analysis including: selecting the concept, determining the purpose of the analysis, identifying all the applications of the concept, determining the defining the attributes of the concept, identifying a model case, identifying related cases, borderline cases and contrary cases, identifying the antecedents and consequences of the concept and finally defining the empirical referents.

### Data sources

**Fig. 1:**
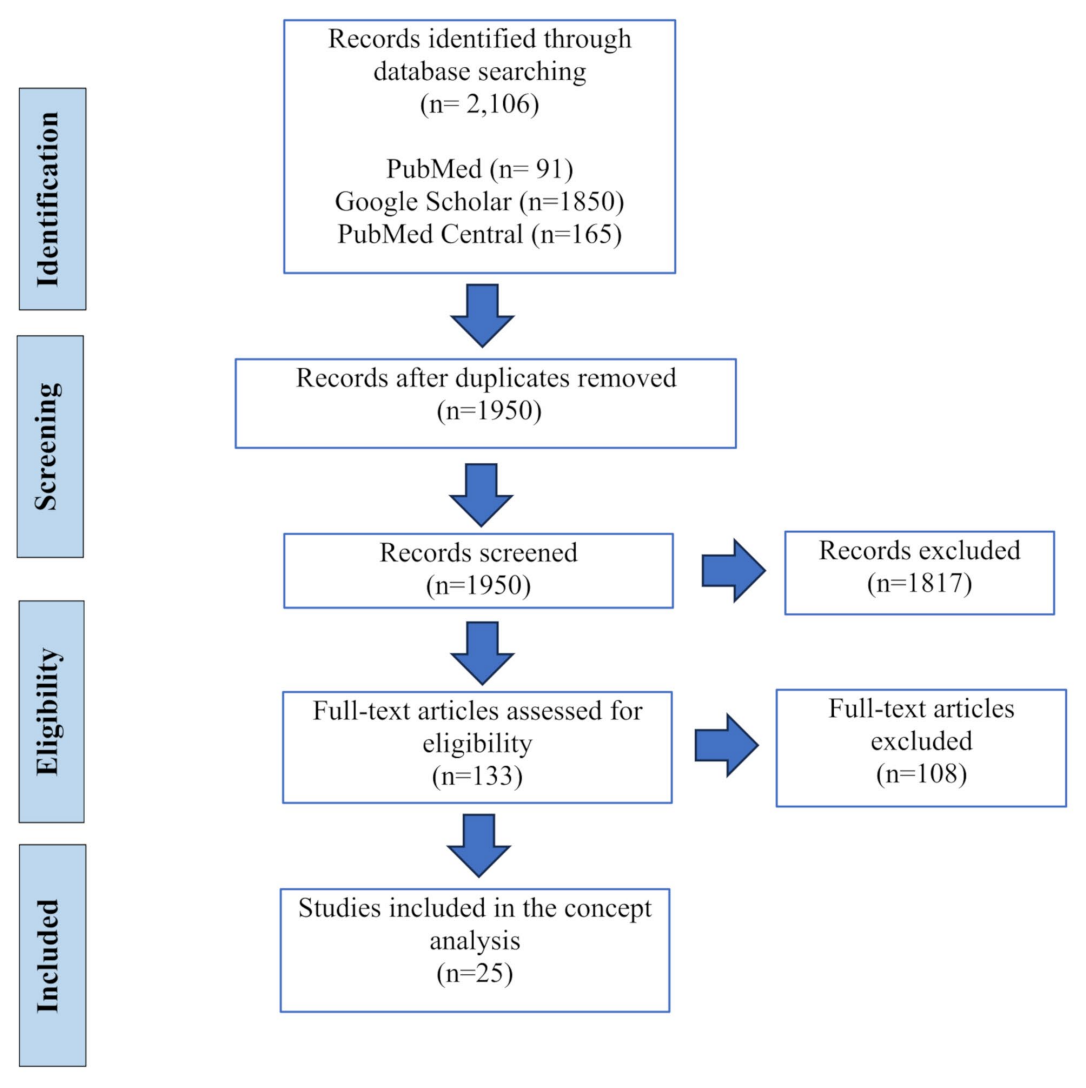
Flow diagram of the screening process and included studies

The review of the literature was conducted using PubMed, PubMed Central, and Google Scholar. Search terms included “translational bioethics” or “practical bioethics” or “nursing bioethics” or “translational ethics”. The search terms were carefully chosen based on commonly used synonyms of translational bioethics identified in preliminary literature review. This strategy ensured we had a comprehensive and efficient retrieval of relevant literature. Initially, a total of 2,106 papers were retrieved. Then, a total of 156 duplicates were identified and removed by Mendeley. After examining the title and abstract of each paper, 1925 papers were excluded based on the eligibility criteria. The eligibility criteria excluded papers that were not in English, unrelated, or the definitions of TB were not reported. Additionally, studies that did not thoroughly engage with the conceptual dimensions of translational bioethics and explore the core elements of the concept were excluded. The papers included were selected because they addressed and thoroughly defined the concept of translational bioethics. Two online dictionaries and 25 papers were included in the concept analysis. Figure 1 presents a flowchart of the screening process.

### Step 1: selection of concept

Following Walker and Avant’s [6] model, the concept of TB was selected due to its growing relevance in bridging the gap between ethical theory and practical application in biomedical contexts. Traditional bioethics has been critiqued for its limited influence on real-world decision-making, highlighting concerns about bioethics being too theoretical [7]. In contrast, TB aligns with the translational science paradigm of moving ethical insights from conceptual development to clinical, regulatory, and societal implementation.

This concept is under-theorized, and critical amid rapid advances in biotechnologies like CRISPR-based gene editing, AI in diagnostics and global health challenges. Considering the fact that nurses are the largest workforce in the health sector, their understanding of the concept can facilitate real world application of ethical insights. The concept of translational bioethics needs to be clearly conceptualized and operationalized to provide nurses with a practical framework for navigating ethical dilemmas in changing clinical environments.

### Step 2: aims of the analysis

The purpose of this concept analysis is to provide clarity and practical meaning to the term TB within the context of nursing. A deeper understanding of TB, its antecedents, defining attributes and consequences, equips nurses to bridge the gap between ethical theory and bedside practice. This will enable them to make informed, context-sensitive decisions.

## Results

This section presents the results from extracting the definitions from the literature retrieved and the analysis of the definitions for defining attributes, antecedents and consequences.

### Step 3: identifying uses of the concept

The Oxford Learner’s Dictionary defines translation as the process of changing something into a different form [8]. The Merriam-Webster Dictionary defines bioethics as a discipline that deals with the ethical implications of biological research and applications especially in medicine [9]. TB has been defined as “the application of legal and ethical principles to research practice or to the consultation room” [10]. It encompassed the It may also be considered as an extension of research ethics that takes into consideration societal impacts of translational research and interdisciplinary collaboration from various disciplines, including social sciences, ethics, law, and public health [11]. Furthermore, it encompasses all types of reasoning and practical conclusions, such as policymaking, that shape real-life medical and environmental decisions [12, 13]. These decisions can involve anything from clinical practice to healthcare interventions decisions [12]. TB works to close the “theory-practice gap” in ethics [7, 14, 15]. This can include guiding nurses and healthcare providers in making ethically sound decisions that reflect real-world challenges. TB is concerned with interventions, changes in practice and policies [7] and has the goal of supporting change in health systems [16]. Furthermore, the translational role of bioethics helps in guiding the refinement of clinical practices in response to ethical concerns such as patient safety [17]. TB is not for ethicists only as it is concerned with translating ethical principles to support non-bioethicists, such as researchers and healthcare practitioners, in dealing with ethical dilemmas [15]. Additionally, TB provides avenue for navigating ethical implications resulting from ongoing scientific and technological developments dilemmas [18]. Nurses, as key healthcare providers, must be able to navigate this complex landscape to ensure ethical, equitable, and high-quality care for patients.

### Step 4: defining attributes

Walker and Avant [6] suggest that recurring qualities of a concept help distinguish it from others. The review of literature revealed four primary defining characteristics of TB.

### Bridging the theory-practice gap

This attribute involves ensuring ethical principles are applied in real-world settings. TB seeks to close the gap between academic scholarship and contemporary policy and practice [19–21]. According to Lanphier et al. (2022), bioethics does not make sense without attention to the real-world practices and the lived realities of healthcare [22]. TB seeks to make a tangible difference by developing practical interventions and supporting healthcare providers including nurses and in adopting bioethical principles [2, 7]. It integrates ethical principles, particularly those focused on safeguarding human participants, into the ethical framework guiding clinical practice [18]. This attribute is therefore crucial as the “theory-practice gap” is a key and foundational problem TB aims to solve.

### Ethical evaluation and decision-making

This involves examining ethical principles and their implementation in translation. High-quality translational bioethics requires attention to context [23]. TB further emphasises the critical consideration of existing real-world bioethical issues [19]. Due the changing nature of healthcare affected by factors like technological advancement and globalization, examining the ethical principles and the context of the occurrence of ethical issues is crucial. TB should convey insights on ethical issues in healthcare practice to audiences outside the traditional field of bioethics [16]. In the practice of translational bioethics, ethicists identify and analyse ethical concerns and arguments, thereby contributing to more informed and reflective decision-making processes within healthcare institutions [24]. In effect, this will help identify if the translation process helped address the initial ethical challenge or resulted in new unintended ethical concerns [25]. A collaborative effort in assessing ethical concerns and providing practical solutions and evaluating the effectiveness of the ethical solution will lead to meaningful outcomes. This attribute also requires the development of institutional policies and guidelines that describe the processes in context-based evaluation and decision-making.

### Interdisciplinary collaboration

Another hallmark of TB is the collaboration between different professionals. It involves cooperation between professionals from various fields such as social sciences, ethics, public health, law, and medicine [11]. Sisk et al. [25] also argue that translating ethical norms into practice requires expertise from multiple disciplines and stakeholders. It is key to addressing the broad societal implications and ensuring that ethical decisions are made from a cross-disciplinary perspective [11, 26]. Nurses must collaborate with ethics committees, legal professionals, and other healthcare experts, as emerging ethical issues are often too complex to be addressed by a single discipline alone.

### Social responsibility and societal impact

This attribute is concerned with making sure the integration of ethical principles into practice makes positive societal impact. Translational bioethics takes into consideration the social implications of bioethics research [17, 27, 28]. When considering translation within practice, some normative claims can be seen as acceptable or not depending on their impact on real-world circumstances [29]. Proponents of TB argue that it takes into account the broader societal consequences that may arise from emerging scientific advancements [26, 30]. Consider the normative claim that “all patients should be informed of incidental findings in genomic research.” While this may seem ethically sound in theory (respecting autonomy), its translation into practice may overwhelm patients with uncertain information, cause unnecessary anxiety, and burden the healthcare system with follow-up requirements. In this case, Bærøe et al. [29] suggest assessing whether this norm, when applied, promotes ethically desirable outcomes overall (in relation to beneficence and justice) rather than strictly adhering to it in all contexts.

These attributes clearly illustrate the necessary activities that must take place for TB to be fully actualized. Embedded in TB are several bioethical activities including policy development, ethical consultation, research, and public engagement.

### Step 5: developing a model case

The model or sample case is a practical example of the concept under study and must contain all of the defining attributes [6]. The fictional model case below demonstrates the defining attributes of translational bioethics.

At a regional hospital, a team of gynecological oncologists and nurses noticed that most cervical cancer patients were presenting at late stages, resulting in high mortality. Data from the hospital revealed that fewer than 5% of women had undergone prior screening. Recognizing this as a serious health equity issue, the team initiated a collaborative effort involving a bioethicist, a public health expert, and community health workers. Together, they reviewed evidence on structural and cultural barriers to screening and critically assessed the hospital’s outreach, education, and service delivery practices. Their ethical analysis, grounded in justice and beneficence, highlighted systemic gaps that disproportionately affected low-income and rural women.

The team co-developed a culturally appropriate intervention that included mobile screening units, multilingual health education, and community engagement through local women’s groups. Hospital administrators supported policy revisions to integrate the program into routine services. The intervention was launched with ongoing evaluation of clinical outcomes and ethical impact. Feedback loops with community members allowed the team to adjust the approach in real time to ensure relevance, fairness, and sustainability.

### Step 6: developing additional cases

Step six of Walker and Avant’s concept analysis involves a definition of other items, which may include borderline, related, contrary, or invented items [6]. A fictional borderline case, related case and one contrary case are discussed here.

### A borderline case

They may contain most or even all of the defining characteristics but differ substantially in one of them [6].

Nurses in a hospital’s intensive care unit (ICU) become concerned about the ethical challenges arising from the use of life-sustaining treatments for patients with very poor prognoses. They initiate discussions among themselves and consult the hospital’s ethics committee. The ethics committee reviews relevant ethical guidelines and legal precedents to develop recommendations for improving communication with families and facilitating shared decision-making about end-of-life care. They implement training sessions for ICU staff on these recommendations.

While this initiative focuses on ethical evaluation and aims to improve practice, it lacks a significant engagement with broader interdisciplinary collaboration beyond the existing hospital structure. The impact of the training on actual changes in practice is not systematically evaluated.

### A related case

Related cases are instances of concepts that are related to the concept being studied but that do not contain all the defining attributes [6].

A group of nurses in a medical department setting is conducting a study on the effectiveness of a new pain management protocol for postoperative patients. They adhere strictly to the ethical guidelines for research involving human subjects, ensuring informed consent and minimizing risks. They collaborate with nurses at surgical departments and statisticians on the research design and data analysis.

The primary focus of this research is on generating research evidence and adhering to research ethics rather than the broader, active translation of existing ethical concepts into widespread changes in healthcare policy or addressing societal implications beyond the immediate research participants.

### A contrary case

Contrary cases are clear examples of “not the concept” [6]. This is a fictional contrary case.

A nurse routinely administers medications to patients without verifying patient identity or allergies, citing being too busy due to understaffing. When questioned by a colleague about the potential risks, the nurse dismisses their concerns and continues the practice. This situation demonstrates a failure to apply basic ethical principles of non-maleficence and fidelity to professional standards. There is no attempt to bridge any gap between ethical theory and practice; instead, there is a disregard for established ethical guidelines.

There is no ethical evaluation, decision-making process, interdisciplinary collaboration, or consideration of social responsibility or societal impact involved.

### Step 7: identifying antecedents and consequences

#### Antecedents

This step helps in identifying the events or occurrences that must have occurred or existed before a concept happens [6]. The literature review revealed significant antecedents to translational bioethics.

1. **Recognizing the Complexity of Issues**: A core precedent to TB is the identification of the complexity of ethical issues. For translation to be completed, it is essential to identify the ethical challenge [12, 25]. Nurses encounter several ethical issues in their duties which include issues surrounding end-of-life decision-making, informed consent, confidentiality and rationing of limited resources. A nurse identifies these ethical issues through comprehensive assessments of individuals, families and communities.
2. **Identifying a Theory-Practice Gap**: Acknowledging that a gap exists motivates the need for translational bioethics to bridge it by translating ethical theories into actionable policies and practices [7]. For TB to take an effect, healthcare providers must acknowledge the need to translate ethics into practice. Nurses through comprehensive assessments of clients and communities may identify ethical concerns such as health inequalities and cultural insensitivity in care delivery. The attributes of TB can then be applied to address these concerns.
3. **Commitment to Real-World Impact**: TB seeks to bride the theory-practice gap and hence demands a commitment to make impact. The main motivation for translational bioethics would have to be practical influence, to create change in the real world [7]. Nurses, in their daily duty execution, embody this commitment through a variety of actions that extend beyond routine clinical care.
4. **Shared Goals and Values**: TB requires effective interdisciplinary collaboration. For this to be effective, there is the need for shared goals and values among the various participating healthcare professionals. When professionals from various fields agree on shared priorities, collaboration becomes more natural and effective [31]. In doing this, nurses play a vital role as communicators, coordinators, and advocates within the healthcare team. They contribute their unique perspective gained from close and continuous patient interaction, which allows them to highlight ethical concerns that may not be visible to other professionals.
5. **Institutional and Policy Support**: Institutions and policies that encourage interdisciplinary collaboration provide essential antecedents. For example, academic institutions and research centers may establish interdisciplinary research teams or create joint programs that foster collaboration across disciplines [31].
6. **Availability of Ethical Tools and Guidelines**: These tools provide the necessary resources for professionals to make ethical decisions in complex situations [2, 24]. Translational activities require self-reflexivity and ethical justification [29]. The availability of these resources is a prerequisite for effectively navigating these challenges. Structured ethical frameworks, institutional policies supporting ethical deliberation (such as ethics committees or decision-making protocols), and ethical case discussions are key strategies that guide teams through complex scenarios and facilitate collaborative ethical decision-making [32].

#### Consequences

Walker and Avant [6] described consequences as the events or incidents that occur as a result of the occurrence of the concept. The outcomes of TB are diverse.

**Fig. 2:**
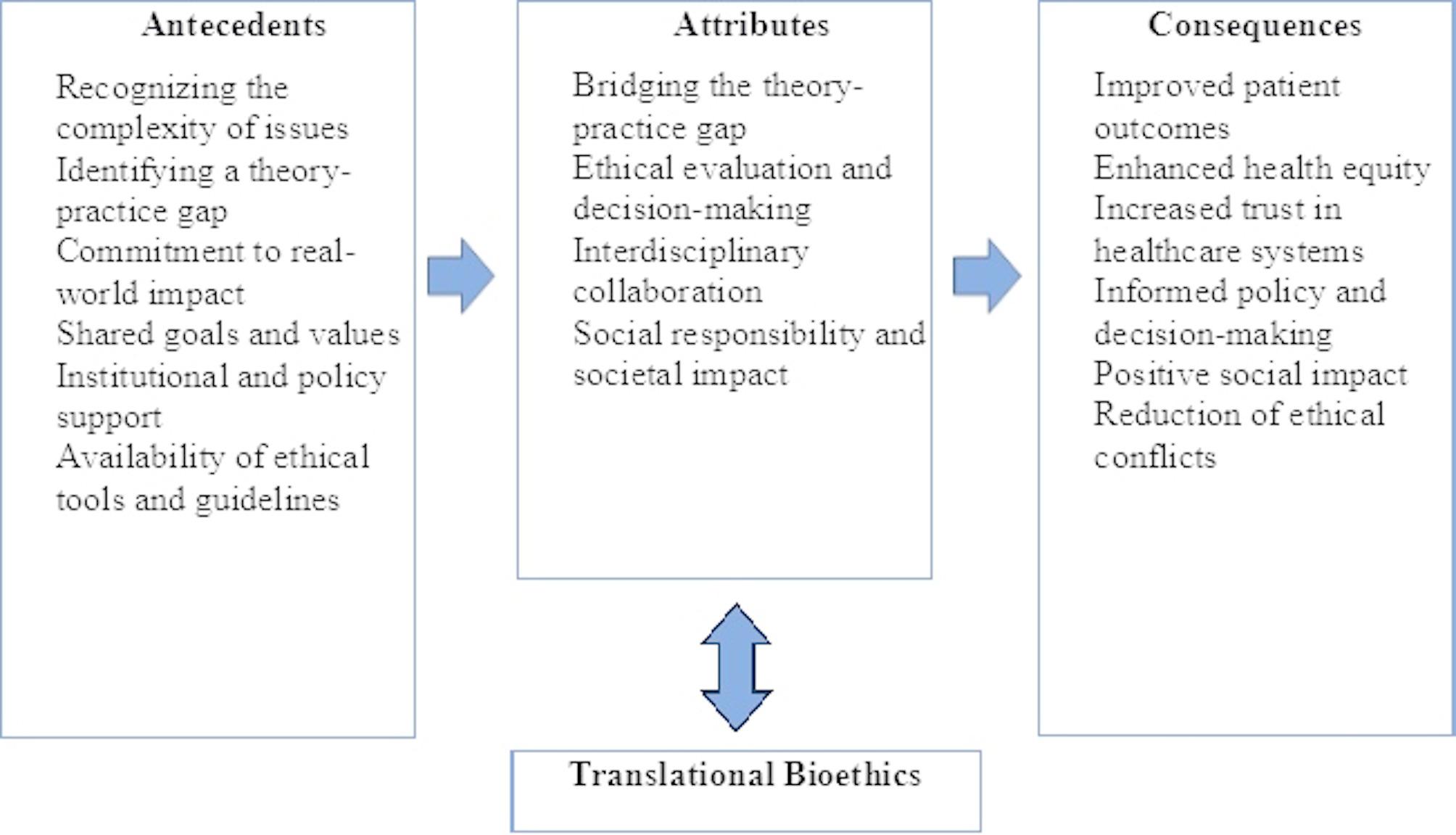
Conceptual model of antecedents, attributes, and consequences of translational bioethics

1. **Improved Patient Outcomes**: By translating ethical principles into practice, TB leads to better patient outcomes. A model application of TB resulted in mortality reduction in patients with acute ST Elevated Myocardial Infarction [1]. With such an end-goal in sight, nurses can effectively contribute to TB by integrating ethical reasoning into everyday care decisions. Through that, nurses play a pivotal role in transforming ethical principles into measurable improvements in patient health and well-being.
2. **Enhanced Health Equity**: The goal of TB is to address fundamental societal issues, including the effects of translational science on public health, health equity, and human flourishing [31]. Majumder et al. [30] illustrated enhanced equitable access to quality care in SCD when viewed under the lens of translational bioethics.
3. **Increased Trust in Healthcare Systems**: TB has a core principle of making positive social impact. When that is achieved, the trust in healthcare systems increases. Evans [27] demonstrated how using the concept might lead to support for research on human brain organoids. Translational bioethics can therefore increase public trust in healthcare systems and bioethical decision-making processes.
4. **Informed Policy and Decision-Making**: TB helps policymakers by providing evidence-based ethical guidelines that shape healthcare policies and practices [7, 13, 16, 25]. Case studies of TB serve as reference points for decision makers.
5. **Positive Social Impact**: By integrating public values and addressing the societal implications, translational bioethics can lead to positive social changes. Boyle et al. [33] based on ethical principles of beneficence, nonmaleficence, autonomy, and justice to develop a significant protocol for a labor and delivery unit during the early phase of the COVID-19 pandemic. This study resulted in the development of significant protocols in a real-world crisis [33].
6. **Reduction of Ethical Conflicts**: Translational bioethics helps minimize conflicts in clinical settings by providing clear ethical frameworks for decision-making. It helps in resolving with cases with ethical dilemma [34]. The attributes of TB such as interdisciplinary collaboration and ethical evaluation ensure all ethical issues are resolved in the application of TB. Figure 2 presents a conceptual model of the antecedents, attributes, and consequences of TB.

### Step 8: empirical referents

This involves the verification of the occurrence of the concept of TB. They are categories of actual phenomena that by their existence or presence demonstrate the occurrence of the concept itself [6].

1. **Development and Implementation of Ethical Guidelines**: When TB is implemented successfully, there will be clear evidence of the application of ethical guidelines. Many hospitals and institutions have ethical committees that ensure ethical practice and this further ascertains a way through which TB happen. Studies that have applied TB have demonstrated this empirical referent [1, 27, 30, 33]. Crico et al. [35] found that healthcare workers including physicians and nurses perceive clinical ethic committees as helpful and valuable in improving the quality of care.
2. **Client satisfaction** – TB occurrence can be examined through the lens of patient satisfaction. Evaluating user satisfaction helps healthcare workers in assessing the practical impact of bioethical interventions on healthcare delivery. The SERVQUAL (service quality) instrument has been a preferred tool in many satisfaction studies [36]. Nurses can conduct patient satisfaction surveys after implementing TB to evaluate its effectiveness.
3. **Case Studies Illustrating Ethical Decision-Making**: Practical application of TB can be found in case studies, which represent concrete examples of how ethical principles are translated into real-life healthcare decisions and outcomes. TB has been documented in management of ST elevated myocardial infarction [1] and advanced cervical cancer [34].
4. **Policy Reforms Influenced by Bioethical Research**: Legislative or institutional policy changes that result from bioethical studies serve as empirical referents. Mertz et al. [37]​ found 17 applications of bioethical research which included support for policy making, making recommendations on how to address ethical challenges, and pleading for stricter application of existing rules.

## Proposed definition

Based on the present analysis, translational bioethics can be defined as an approach that bridges ethical theory and practice through research, interdisciplinary collaboration, and a focus on contextual ethical evaluation and decision making, aiming for socially responsible and impactful outcomes.

## Discussion

Translational bioethics is an emerging field focused on bridging the gap between biomedical research and clinical practice. The core of TB lies in its aim to move beyond abstract ethical theory and ensure practical application in real-world contexts [2, 7, 21]. The field of bioethics is complex and the concept of TB has varied uses across the literature. To apply it to nursing practice, a consistent definition is needed for clarity. Using the Walker and Avant [6] method for completing a concept analysis, four essential attributes were identified. A preliminary definition was then proposed based on these findings.

A core component of TB is the evaluation of ethical interventions and tools. Nurses can involve TB by conducting research to identify ethical principles and guidelines before implementing proposed actions or changes. According to Sisk et al. [25], this evaluation process helps in determining the significance of the translation process in addressing ethical challenges. This is crucial in nursing practice because of the confrontations with ethical issues nurses face.

TB has been demonstrated to bridge the theory-practice gap. Parsons et al. [21] posit that translational bioethics bridges the gap between academic reflection and current policy and practice. The activities of nursing are integral to healthcare and it is relevant to understand that nursing activities should encompass implementing theoretical knowledge to practice.

Interdisciplinary collaboration plays a major role in translational bioethics. The use of multidisciplinary approach results in generating ideas, knowledge, concepts, methodologies, experience and instruments to comprehensively solve a common problem [1]. Nurses play important roles in multidisciplinary teams. Alanazi et al. [32] illustrated some functions of nurses working in a multidisciplinary team as focusing on holistic care, including patient comfort and emotional support.

## Implications for nursing practice

The importance of translational bioethics in nursing stems from its ability to guide researchers and clinicians in navigating ethical dilemmas [18]. It ensures that ethical considerations are integrated into the research and practice for public impact. Nurses should integrate translational bioethics into their practice by actively engaging with ethical challenges through structured reflection, participation in ethics consultations, and incorporation of evidence-based ethical frameworks into clinical decision-making. This integration is feasible across various nursing roles, including bedside practice through ethical reflection, leadership through policy development, and research or education through the application of ethical insights. It supports ethical patient care while advancing broader outcomes such as improved care quality, greater equity, and strengthened moral agency within the profession.

## Ethical statement

This concept analysis did not need ethical approval because it did not entail gathering original data from either human or animal subjects. The study followed the rules of responsible research conduct and academic integrity and was fully founded on a thorough examination of the body of current literature.

## Limitations

This concept analysis has some limitations. To begin with, only articles published in English were included in the analysis, and this potentially limits other perspectives of translational bioethics. It is therefore recommended that future studies explore more diverse cultures. In addition, the analysis may be subject to some biases such as selection bias, analysis bias and extraction bias. This was tackled by extensive review of papers and discussion of extracted data.

## Conclusion

The analysis revealed a clarified definition of the concept, its attributes, antecedents and the consequences of successful TB. TB is a crucial and evolving field that seeks to make ethical principles actionable and relevant in the complexities of modern healthcare. Its implicit presence in discussions around various healthcare concepts and nursing underscores its fundamental importance in ensuring ethical, equitable, and high-quality care for patients across all domains of practice, including nursing. Clinicians, including nurses can base on the attributes of TB to implement actions that will make patient-provider interactions more professional, ethical, and effective leading to consequences such as improved patient outcomes, enhanced health equity, increased trust in healthcare systems and positive social impact.

## Data Availability

The datasets analysed during the current study are available in the Open Science Framework repository via the link https://osf.io/64zxd/?view_only=46fc7df4d66b4f1abfb5307fdabff383.
